# *Coccomyxagreatwallensis* sp. nov. (Trebouxiophyceae, Chlorophyta), a lichen epiphytic alga from Fildes Peninsula, Antarctica

**DOI:** 10.3897/phytokeys.110.26961

**Published:** 2018-11-02

**Authors:** Shunan Cao, Fang Zhang, Hongyuan Zheng, Fang Peng, Chuanpeng Liu, Qiming Zhou

**Affiliations:** 1 Key Laboratory for Polar Science SOA, Polar Research Institute of China, No.451 Jinqiao Road, Pudong Avenue, Shanghai, 200136, China Polar Research Institute of China Shanghai China; 2 College of Environmental Science and Engineering, Tongji University, Shanghai 200092, China Tongji University Shanghai China; 3 China Centre for Type Culture Collection (CCTCC), College of Life Sciences, Wuhan University, No. 299 Bayi Road, Wuchang District, Wuhan 430072, China Wuhan University Wuhan China; 4 School of Life Science and Technology, Harbin Institute of Technology, 2 Yikuang Street, Nangang Distinct, Harbin, 150080, China Harbin Institute of Technology Harbin China

**Keywords:** Lichen epiphyte, Morphology, TEM, Phylogeny

## Abstract

A single-celled green alga *Coccomyxagreatwallensis* Shunan Cao & Qiming Zhou, **sp. nov.**, isolated from a specimen of Antarctic lichen *Psoromahypnorum* (Vahl) Gray, is described and illustrated based on a comprehensive investigation of morphology, ultrastructure, ecology and phylogeny. The cells of *C.greatwallensis* are ovoid to long ellipsoidal and measured 3–5 µm × 6–12 µm. The new species has distinct ITS rDNA and SSU rDNA sequences and differs from the phylogenetic closely related species *C.antarctica*, *C.arvernensis* and *C.viridis* in cell size, distribution and habitat.

## Introduction

The coccoid green algal genus *Coccomyxa*Schmidle (1901) is well known for its diversified ecological habitats and worldwide distribution. Algae of this genus has been reported as free living (Blanc et al. 2012), endophytic (Tremouillaux-Guiller et al. 2002; Zuykov et al. 2014) and lichen photobionts (Zoller and Lutzoni 2003; Muggi et al. 2010). *Coccomyxa* can survive under extremely harsh environments, such as in the spent fuel cooling pond of a nuclear reactor (Rivasseau et al. 2016), in a highly acidic lake (pH~2.6) (Hrdinka et al. 2013), as well as in polar regions (as low as -88 °C) (Blanc et al. 2012).

Based on the mucilaginous colonies’ structure, cell length and width variability details, Jaag (1933) delimited 33 species of this genus, including 14 free-living, 13 lichenised and 6 lichen epiphytic species. Subsequently, only seven species were well recognised based on some morphological characters, such as cell shape and size, chloroplasts numbers and mucilage properties (Ettl and Gärtner 1995). However, the morphological characters of *Coccomyxa* depend on the culture conditions, for example, salinity influenced the phenotypic plasticity significantly (Darienko et al. 2015) and nutrient availability influenced the presence of mucilaginous sheaths (Malavasi et al. 2016). As the instability of morphological features led to a problematic morphological delineation of the genus *Coccomyxa*, a DNA-barcode based method has been developed and seven distinct species were subdivided (Darienko et al. 2015). Malavasi et al. (2016), combining morphological characters, ecological features and DNA sequences of *Coccomyxa*, recognised 27 species scenarios. Subsequently, *Coccomyxaantarctica* Shunan Cao & Qiming Zhou, 2018 was described as a new epiphytic species living with lichen *Usneaaurantiacoatra* on King George Island (Cao et al. 2018)

Currently, 28 species scenarios have been accepted, amongst which *C.actinabiotis* Rivasseau, Farhi & Couté, 2016, *C.antarctica*, *C.avernensis* Jaag, 1933, *C.polymorpha* T. Darienko & T. Pröschold, 2015, *C.subellipsoidea* E. Acton, 1909 and undescribed *Coccomyxa* spp., belonging to Clade I, Clade KL and Clade N according Malavasi et al. (2016), are the eight epiphytic species scenarios. Meanwhile, the species *C.avernensis* and *C.subellipsoidea* are also reported as lichen photobionts. The other lichenised species scenarios include *C.disper* Schmidle, 1901, *C.solorinae* Chodat, 1909, *C.viridis* Chodat, 1913 and *Coccomyxa* Clades A, D and F (Malavasi et al. 2016). The species *C.antarctica*, *C.dispar*, *C.subellipsoidea* and *C.simplex* Mainx, 1928 show the Antarctic distribution, amongst which *C.simplex* is the only free living one (Holm-Hansen 1964; Darienko et al. 2015; Borchhard et al. 2017; Cao et al. 2018).

The Fildes Peninsula undergoes a typical sub-Antarctic oceanic climate with relatively high precipitation (89%) with 56–64 mm rainfall, wind blowing from west through northwest with a speed of 6.8–7.4 m/s and the average temperature ranging from 0.5–1.8 °C in summer (Yang et al. 2013). About 127 lichen species have been recorded in Fildes Peninsula (http://www.aari.aq/KGI/Vegetation/lst_lichens.html). The lichen *Psoromahypnorum* (Vahl) Gary, one of the four *Psoroma* spp. found in this region, is characterised by its squamulose thallus without secondary products, dull brown discs, apothecia margin without or with very short hairs (Øvstedal and Smith 2001). Both cyanobacteria and green algae have been reported as photosynthetic partners of *P.hypnorum* (Holien and Jørgensen 2000; Øvstedal and Smith 2001; Wirtz et al. 2003; Ekman et al. 2014).

In the current study, a lichenicolous single cell green alga was isolated from *P.hypnorum*. Based on the comprehensive analysis approach, including morphology, ultrastructure, ecology and phylogeny, the green alga is demonstrated to be new to science.

## Material and methods

### Isolation and culture

The lichen specimen (collection No. 274) of *Psoromahypnorum* was collected from Fildes Peninsula, King George Island, Antarctica (62°12.69'S, 58°55.70'W) during the 30^th^ Chinese National Antarctic Research Expeditions in summertime (1 February 2014–15 March 2014). The specimen was kept in the Resource-sharing Platform of Polar Samples which includes samples of Biology, Ice-snow, Rock, Deep-space and Sediment (BIRDS ID 2131C0001ASBM100076) at 4 °C till the isolation was processed.

A single algal cell was obtained following a modified aseptic isolation procedure (Cao et al. 2018). The isolations, cultured on a petri-dish with PDA and BBM medium in an illumination incubator (4 °C, 12 hr light/12 hr dark), were deposited in the Freshwater Algae Culture Collection at the Institute of Hydrobiology (FACHB) as an open collection (FACHB-2139).

### Light and electron microscopy

For observing and photographing the algal cultures, compound microscopes Nikon Eclipse 80i and Nikon ACT-1 V2.70 were used.

After fixing with 2.5% glutaraldehyde buffer, the algal cells were used for transmission electron microscopy (TEM). The procedures and reagents (including 2.5% glutaraldehyde buffer) used followed Cao et al. (2018). The 70 nm cell sections, cut by a Leica EM UC6 ultramicrotome and stained with 3% uranyl acetate and lead citrate, were observed using a Jeol JEM1230 transmission electron microscope at 80–120 kV. The micrographs were captured using iTEM software by an Olympus SIS VELETA CCD camera.

### DNA extraction, amplification, sequencing and analysis

A modified CTAB method (Cao et al. 2015) was used to extract the alga genomic DNA. Primer pairs NS1, NS4; NS3, NS6; NS5, NS8 (White et al. 1990) and primer pair ITS5, O2 (Cao et al. 2015) were used to amplify the SSU rDNA and ITS rDNA, respectively. A 50 µl volume PCR reaction was selected, PCR application and products verification followed Cao et al. (2015) and double-stranded PCR products were sequenced by an ABI3730XL sequencer.

SEQMAN programme within Lasergene v.7.1 software (DNASTAR Inc.) was selected to check the double-directional ITS rDNA and SSU rDNA sequences. These two regions were overlapped into one single contig and the flanking regions were trimmed off. The sequence representing the new species was submitted to GenBank (MF465899).

ClustalW algorithm, including in MEGA 7 (Kumar et al. 2016), was performed to align the sequences with default parameters (Higgins et al. 1994) and then adjusted manually. The Neighbour-Joining (NJ) was selected to calculate the ITS phylogenetic structures, as well as Maximum Likelihood (ML) method for SSU sequences. Pairwise distances of ITS rDNA and SSU rDNA sequences were calculated using MEGA 7. A 1000 resamplings bootstrap was tested for the reliability of the inferred trees. In total, 42 sequences, which have been confirmed by Malavasi et al. (2016), were retrieved from GenBank (Table 1).

**Table 1.** *Coccomyxa* spp. sequences used in the present study.

Species	Collection No.	GenBank No.	
		ITS rDNA	SSU rDNA
Clade B*Coccomyxa sp.	GA5a	AB917140	AB917140
Clade D*Coccomyxa sp.	CCAP 216/24	FN298927	FN298927
	CCAP 812/2A	HG972992	HG972992
Clade E*Coccomyxa sp.	IB-GF-12	–	KM020052
Clade E*Coccomyxasubellipsoidea	CCAP 812/3	HG972972	HG972972
Clade H*Coccomyxa sp.	KN-2011-U5	HE586557	–
Clade I*Coccomyxa sp.	KN-2011-T3	HE586515	HE586515
	KN-2011-T1	HE586550	–
Clade K*Coccomyxa sp.	KN-2011-C4	HE586508	HE586508
Clade L*Monodus sp.	UTEX B SNO83	–	HE586506
Clade M*Monodus sp.	CR2-4	HE586519	HE586519
Clade N*Coccomyxaviridis 3	CAUP H5103	HG973007	HG973007
	SAG 2040	HG973004	HG973004
* Coccomyxa actinabiotis *	216-25	FR850476	FR850476
	KN-2011-T4	HE586516	HE586516
* Coccomyxa antarctica *	FACHB-2140	MF465900	MF465900
* Coccomyxa arvernensis *	SAG 216-1	–	HG972999
	Wien C19	HG973000	HG973000
* Coccomyxa dispar *	SAG 49.84	HG972998	HG972998
* Coccomyxa elongata *	CAUP H5107	HG972981	HG972981
	SAG 216-3b	HG972980	HG972980
* Coccomyxa galuniae *	CCAP 211/97	FN298928	FN298928
	SAG 2253	HG972996	HG972996
***Coccomyxagreatwallensis* sp. nov.**	**FACHB-2139**	**MF465899**	**MF465899**
* Coccomyxa melkonianii *	SCCA048	KU696488	KU696488
* Coccomyxa onubensis *	ACCV1	HE617183	HE617183
* Coccomyxa polymorpha *	CAUP H5101	HG972979	HG972979
	KN-2011-T2	HE586514	HE586514
* Coccomyxa simplex *	CAUP H 102	HE586504	HE586504
	SAG 216-2	HG972989	HG972989
* Coccomyxa solorinae *	SAG 216-12	HG972987	HG972987
	SAG 216-6	HG972988	HG972988
* Coccomyxa subellipsoidea *	SAG 216-7	HG972976	HG972976
	Wien C20	HG972975	HG972975
	CAUP H5105	HG972974	–
* Coccomyxa vinatzeri *	ASIB V16	HG972994	HG972994
* Coccomyxa viridis *	SAG 216-14	HG973002	HG973002
	SAG 216-4	HG973001	HG973001
* Elliptochloris bilobata *	SAG 245.80	HG972969	HG972969
* Hemichloris antarctica *	SAG 62.90	HG972970	HG972970

* Clades referred after Malavasi et al. (2016).

## Results

### Coccomyxa greatwallensis

Taxon classificationPlantaeChlamydomonadalesCoccomyxaceae

Shunan Cao & Qiming Zhou sp. nov.

Figures 1 , 2

#### Holotype.

**Figure 1.**
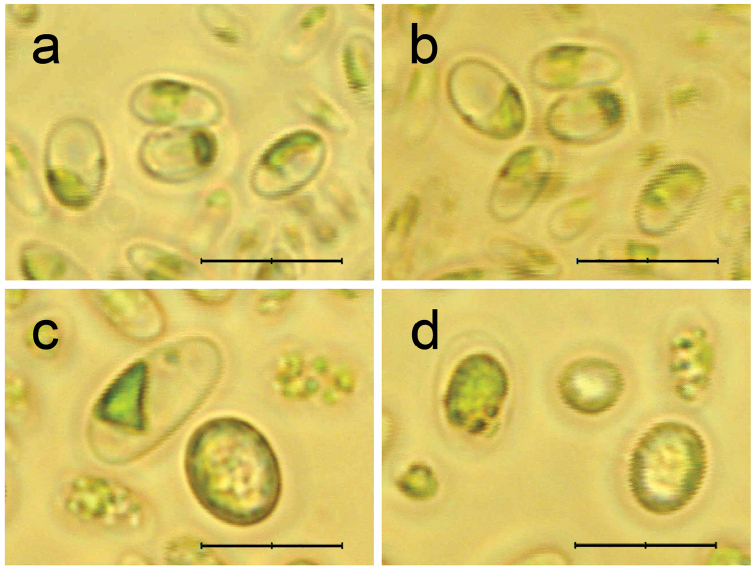
*Coccomyxagreatwallensis* Shunan Cao & Qiming Zhou, sp. nov., light microphotographs. Cells cultured in BBM medium (**a, b**) and in PDA medium (**c, d**). Scale bar: 10 µm.

Strain FACHB-2139, Freshwater Algae Culture Collection, the Institute of Hydrobiology (FACHB-Collection) (Fig. 1a).

#### Type locality.

Antarctic, Fildes Peninsula, on soil (62°12.69'S, 58°557.70'W), 40 m a.s.l.; isolated from the Antarctic lichen *Psoromahypnorum* (collection No. 274, BIRDS ID: 2131C0001ASBM100076) on 14 February 2014.

#### Habitat.

Epiphytic green alga, living with lichen *Psoromahypnorum*in Sub-Antarctic climate.

#### Description.

**Figure 2.**
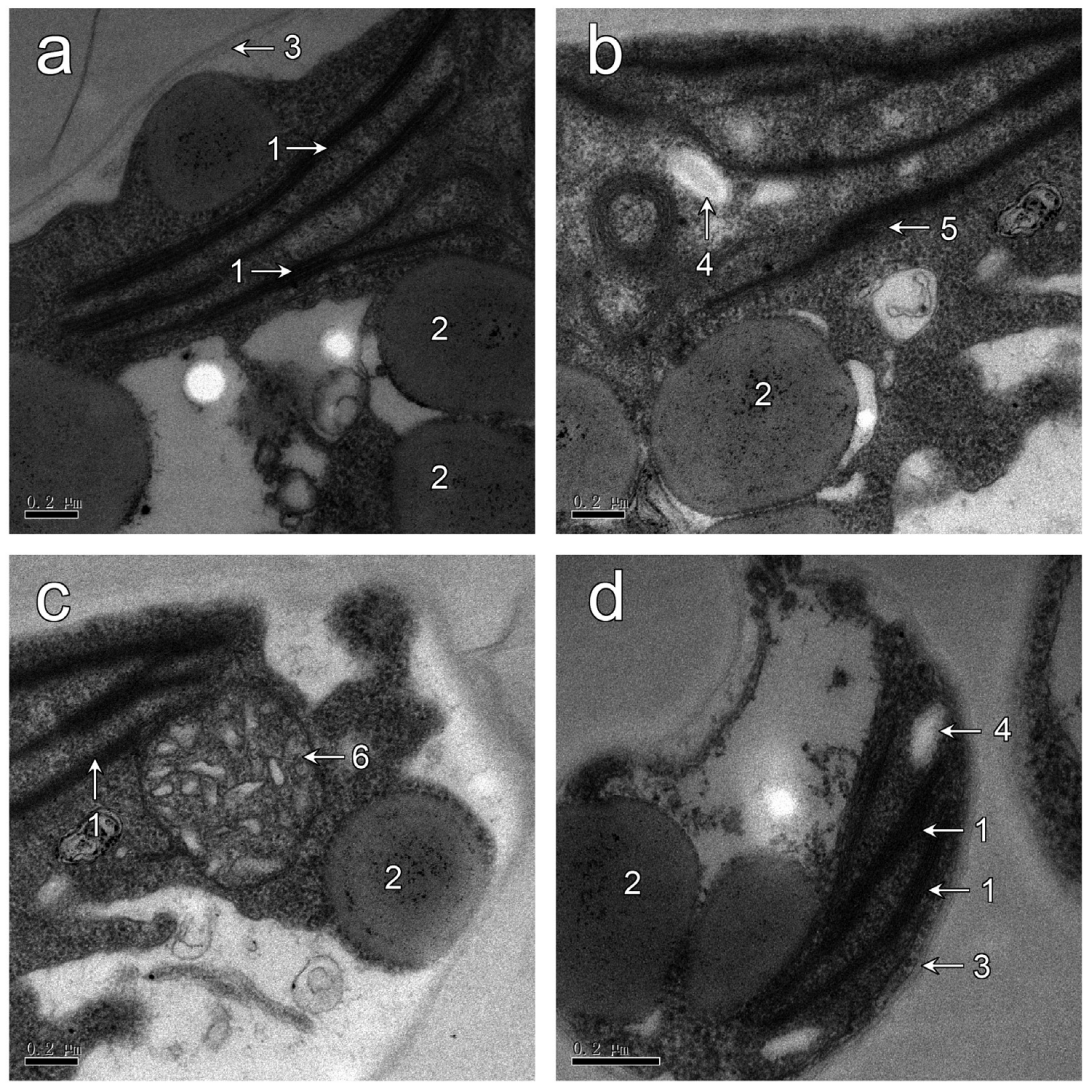
Ultrastructure of *Coccomyxagreatwallensis* Shunan Cao & Qiming Zhou, sp. nov. in PDA medium; note: chloroplast (2), plastoglobuli (2), cell wall (3), starch granules (4), thylankoids (5) and mitochondria (6). Scale bar: 0.2 µm.

Single-celled green alga, ovoid to long ellipsoidal, asymmetrical, measured 3–5 µm × 6–12 µm, some cells nearly rounded in nutrient-rich PDA medium; cells without mucilaginous sheath (Fig. 1). Cell wall smooth, three layers in ultrastructures. Protoplast filled with lipid droplets. Chloroplast parietal, without pyrenoid and with starch granules in the inter thylacoidal spaces. One nucleus in the central part of the cell present. Reproductive process not observed (Fig. 2).

## Molecular analyses

The pairwise distance analysis of ITS rDNA sequences shows that the overall mean distance is 0.171±0.015. The pairwise distance between our algal strain FACHB-2139 and the other species of *Coccomyxa* ranged from 0.253 to 0.022, of which *C.arvernensis* shows the minimum distance with our isolate of 0.022 followed by *Coccomyxa* sp. Clade N of 0.030 (Suppl. material 1: Table S1). The pairwise distance analysis of SSU rDNA sequences shows that the overall mean distance is 0.017±0.002. In addition, the pairwise distance between alga strain FACHB-2139 and the other species of *Coccomyxa* ranged from 0.025 to 0.001, amongst which both *C.arvernensis* and *C.viridis* show the minimum distance of 0.001 with our sample (Suppl. material 1: Table S1). That indicated that alga FACHB-2139 is closely related to *C.arvernensis* and *C.viridis*.

**Figure 3.**
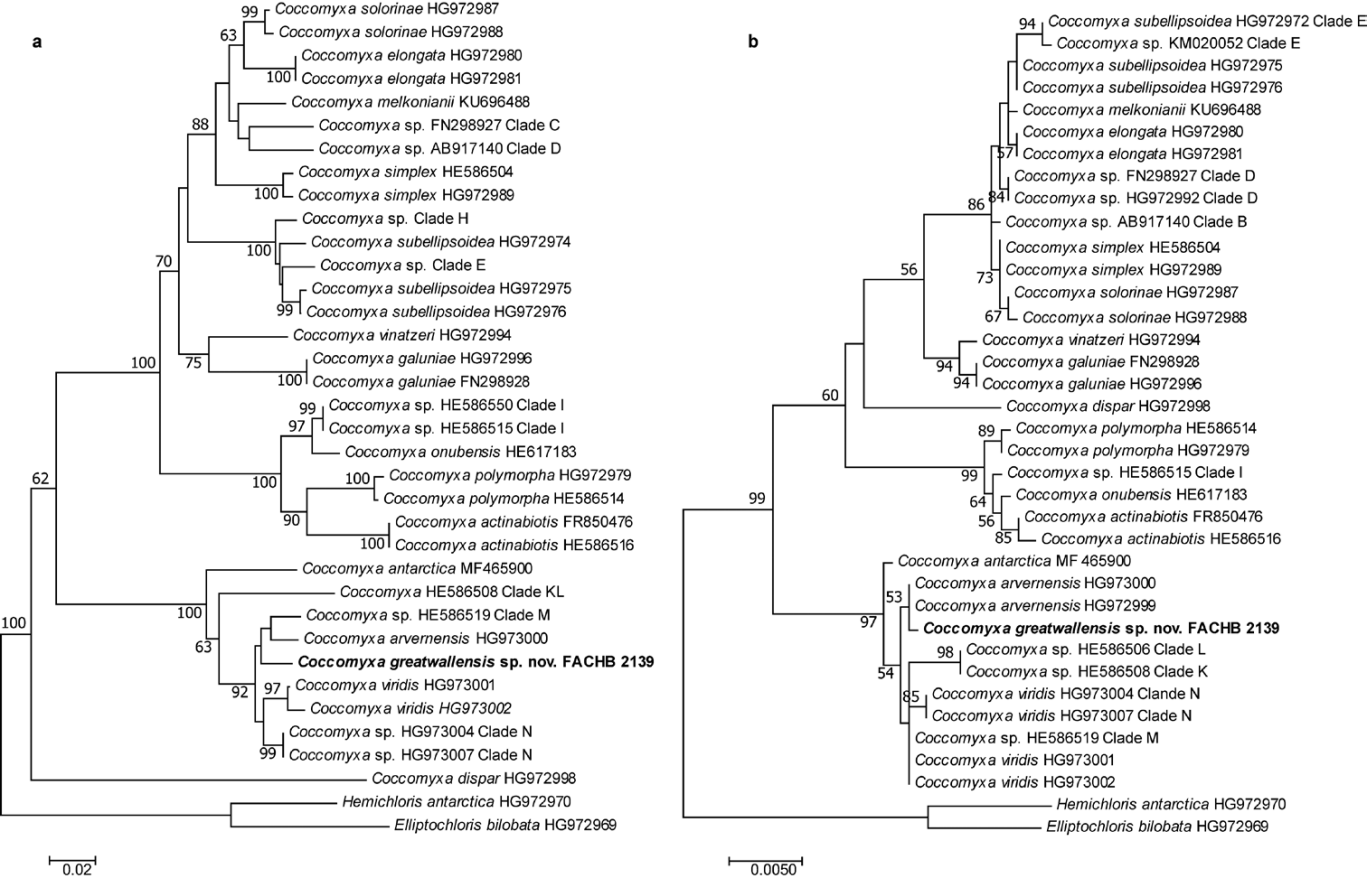
The NJ tree based on ITS rDNA (**a**) and the ML tree based on SSU rDNA (**b**) sequences phylogenetic analyses. The sequences marked with *Coccomyxa* clade A–N referred after Malavasi et al. (2016).

For the ITS rDNA, all the *Coccomyxa* sequences clustered into one group supported with bootstrap value 100 and within *Coccomyxa*, six subgroups have been clustered. The alga FACHB-2139 together with *C.antarctica*, *C.arvernensis*, *C.viridis*, *Coccomyxa* spp. of clade KL, Clade M and Clade N clustered as a subgroup, were supported with a bootstrap value 100; but the newly isolated strain FACHB-2139 differs from the other species clearly, no well supported clade for FACHB-2139 and species mentioned above were formed (Fig. 3a). In the SSU rDNA phylogenetic result, the sequences of *Coccomyxa* clustered into five subgroups and the alga FACHB-2139, *C.antarctica*, *C.arvernensis*, *C.viridis*, *Coccomyxa* spp. Clade K, Clade L, Clade M and Clade N lay in the same subgroup whose bootstrap value was 97. Though alga strain FACHB-2139 and *C.arvernensis* formed a monophyletic group, this clade was supported by a low bootstrap value 53, which indicated that these two species were insufficiently supported statistically (Fig. 3b).

## Diagnosis

Morphologically, our sample FACHB-2139 can be distinguished from its phylogenetically close congeners *C.viridis* (1.8–3.6 µm × 4.7–8.4 µm) and *C.arvernensis* (3–4 µm × 6–8 µm) (Müller 2005, Hodač 2015) by its larger cells and from *C.antarctica* (4–7 µm × 8–12 µm) by its smaller cells (Cao et al. 2018). The cell sizes of the above species were recorded when cultured in BBM medium. In addition, both *C.viridis* and *C.arvernensis* are lichenised or are epiphytic species and have not been recorded as an Antarctica distribution.

Our molecular and morphological analyses indicate that algal isolate FACHB-2139 represents a new *Coccomyxa* species which we named *Coccomyxagreatwallensis* Shunan Cao & Qiming Zhou sp. nov.

## Discussion

*Coccomyxagreatwallensis* Shunan Cao & Qiming Zhou sp. nov., isolated from Antarctic squamulose lichen *P.hypnorum*, is one of the *Coccomyxa* species, which is characterised by ovoid to ellipsoidal single cells. The usage of molecular barcode provides an effective and stable tool to identify and classify the species of *Coccomyxa* (Darienko et al. 2015; Malavasi et al. 2016). In the current study, both ITS and SSU rDNA were used and a comparison with the closely related species had been listed in Suppl. material 3:Table S3. The minimum pairwise distance was calculated between *C.arvernensis* and *C.greatwallensis* using ITS rDNA sequences, but the bootstrap value, which was lower than 50, did not support these two species as a monophyletic group. A similar result was also obtained using SSU rDNA sequences.

Though some *Coccomyxa* species could be the photosynthetic partner of lichens (Honegger and Brunner 1981), due to the lichen mycobiont’s selectivity to its photobiont partner, one photobiont group occurs within relative stable lichen groups (Tschermak-Woess 1988; Cao et al. 2015); for example, *Coccomyxa* is known as the photobiont of lichenised ascomycots belonging to Peltigerales (i.e. *Nephroma* Müll. Arg., *Peltigera* Willd. and *Solorina* Ach.), Baeomycetales (*Baeomyces* Pers., *Dibaeis* Clem., *Orceolina* Hertel and *Placynthiella* Gyeln.), Pertusariales (*Icmadophila* Trevis.), Agaricales (*Lichenomphalia* Redhead, Lutzoni, Moncalvo & Vilgalys), Lecanorales (*Micarea* Fr.) and Cantharellales (*Multiclavula* R.H. Petersen) (Poulsen et al. 2001; Smith et al. 2009; Wirth et al. 2013), as well as the basidiomycots belonging to Agaricales (*Omphalina* Quél.) (Jaag 1933; Zoller and Lutzoni 2003). In addition, *Coccomyxa* is optionally lichenised with the fungus *Schizoxylonalbescens* Gilenstam, H. Döring & Wedin (Ostropales) (Muggi et al. 2010). Furthermore, there is also evidence to support the photosynthetic partner of Antarctic lichen *P.hypnorum* is cyanobacteria or the green algae*Myrmecia* Printz (Brodo et al. 2001; Øvstedal and Smith 2001; Wirtz et al. 2003; Ekman et al. 2014) but not the species of *Coccomyxa*. We therefore conclude that the newly described green alga *C.greatwallensis* is an epiphytic alga of lichen *P.hypnorum*.

## Supplementary Material

XML Treatment for Coccomyxa greatwallensis

## Supplementary materials

Supplementary material 1 Table S1. Pairwise distance calculated using ITS rDNA sequences Data type: molecular data This is an open access article distributed under the terms of the Creative Commons Attribution License (CC BY 4.0), which permits unrestricted use, distribution, and reproduction in any medium, provided the original author and source are credited.Shunan Cao, Fang Zhang, Hongyuan Zheng, Fang Peng, Chuanpeng Liu, Qiming Zhou

Supplementary material 2 Table S2. Pairwise distance calculated using SSU rDNA sequences Data type: molecular data This is an open access article distributed under the terms of the Creative Commons Attribution License (CC BY 4.0), which permits unrestricted use, distribution, and reproduction in any medium, provided the original author and source are credited.Shunan Cao, Fang Zhang, Hongyuan Zheng, Fang Peng, Chuanpeng Liu, Qiming Zhou

Supplementary material 3 Table S3. Comparison of the closely related species Data type: species data This is an open access article distributed under the terms of the Creative Commons Attribution License (CC BY 4.0), which permits unrestricted use, distribution, and reproduction in any medium, provided the original author and source are credited.Shunan Cao, Fang Zhang, Hongyuan Zheng, Fang Peng, Chuanpeng Liu, Qiming Zhou

## References

[B1] Brodo IW Sharnoff SD Sharnoff S (2001) Lichens of North America. Yale University Press.

[B2] Blanc G Agarkova I Grimwood J Kuo A Brueggeman A Dunigan DD Gumon J Ladunga I Lindquist E Lucas S Pangilinan J Pröschold T Salamov A Schmutz J Weeks D Yamada T Lomsadze A Borodovsky M Claverie JM Grigoriev IV Etten JLV (2012) The genome of the polar eukaryotic microalga *Coccomyxasubellipsoidea* reveals traits of cold adaptation. Genome Biology 13(5): R39. 10.1186/gb-2012-13-5-r39 PMC3446292 22630137

[B3] Borchhard N Schiefelbein U Abarca N Boy J Mikhailyuk T Sipman HJM Farsten U (2017) Diversity of algae and lichens in biological soil crusts of Ardleyand King George islands, Antarctica. Antarctic Science 29: 1–9. 10.1017/S0954102016000638

[B4] Cao SN Zhang F Liu CP Hao ZH Tian Y Zhu LX Zhou QM (2015) Distribution patterns of haplotypes for symbionts from *Umbilicariaesculenta* and *U.muehlenbergii* reflect the importance of reproductive strategy in shaping population genetic structure. BMC Microbiology 15(1): 212. 10.1186/s12866-015-0527-0 PMC4608304 26471277

[B5] Cao SN Zhang F Zheng HY Liu CP Peng F Zhou QM (2018) *Coccomyxaantarctica* sp. nov. from the Antarctic lichen *Usneaaurantiacoatra*. PhytoKeys 98: 107–115. 10.3897/phytokeys.98.25360 PMC5966503 29849474

[B6] Darienko T Gustavs L Eggert A Wolf W Pröschold T (2015) Evaluation the species boundaries of green microalgae (*Coccomyxa*, Trebouxiophyceae, Chlorophyta) using integrative taxonomy and DNA barcoding with further implications for the species identification in environmental samples. PLoS One 10(6): e0127838. 10.1371/journal.pone.0127838 PMC4469705 26080086

[B7] Ekman S Wedin M Lindblom L Jørgensen PM (2014) Extended phylogeny and a revised generic classification of the Pannariaceae (Peltigerales, Ascomycota). Lichenologist (London) 46(05): 627–656. 10.1017/S002428291400019X

[B8] Ettl H Gärtner G (1995) Syllabus der Boden-, Luft- und Flechtenalgen. Spring Spektrum, Berlin-Heidelberg.

[B9] Higgins D Thompson J Gibson T Thompson JD Higgins DG Gibson TJ (1994) CLUSTAL W: Improving the sensitivity of progressive multiple sequence alignment through sequence weighting, position-specific gap penalties and weight matrix choice. Nucleic Acids Research 22(22): 4673–4680. 10.1093/nar/22.22.4673 7984417 PMC308517

[B10] Hodač L (2015) Green algae in soil: assessing their biodiversity and biogeography with molecular-phylogenetic methods based on cultures. Doctor dissertation, Georg-AugustUniversity School of Science.

[B11] Holien H Jørgensen PM (2000) A blue-green *Psoromahypnorum* found in Trøndelag, Central Norway. Graphis Scripta 11: 49–52. 10.2216/i0031-8884-4-1-43.1

[B12] Holm-Hansen O (1964) Isolation and culture of terrestrial and fresh-water algae of Antarctica. Phycology 4(1): 43–52. 10.2216/i0031-8884-4-1-43.1

[B13] Honegger R Brunner U (1981) Sporopollenin in the cell walls of *Coccomyxa* and *Myrmecia* phycobionts of various lichens: An ultrastructural and chemical investigation. Canadian Journal of Botany 59(12): 2713–2734. 10.1139/b81-322

[B14] Hrdinka T Šobra M Fottb J Nedbalová L (2013) The unique environment of the most acidified permanently meromictic lake in the Czech Republic. Limnologica 43(6): 417–426. 10.1016/j.limno.2013.01.005

[B15] Jaag O (1933) *Coccomyxa* Schmidle, Monographie einer Algengattung. Beiträge zur Kryptogamenflora der Schweiz 8: 1–132.

[B16] Kumar S Stecher G Tamura K (2016) MEGA7: Molecular evolutionary genetics analysis version 7.0 for bigger datasets. Molecular Biology and Evolution 33(7): 1870–1874. 10.1093/molbev/msw054 27004904 PMC8210823

[B17] Malavasi V Škaloud P Rindi F Tempesta S Paoletti M Pasqualetti M (2016) DNA-based taxonomy in ecologically versatile microalgae: A re-evaluation of the species concept within the coccoid green algal genus *Coccomyxa* (Trebouxiophyceae, Chlorophyta). PLoS One 11(3): e0151137. 10.1371/journal.pone.0151137 PMC4814044 27028195

[B18] Muggi L Baloch E Stabentheiner E Grube M Wedin M (2010) Photobiont association and genetic diversity of the optionally lichenized fungus *Schizoxylonalbescens*. FEMS Microbiology Ecology 75(2): 255–272. 10.1111/j.1574-6941.2010.01002.x 21133956

[B19] Müller J (2005) Genetic fingerprints of microalgal culture strains: amplified fragment length polymorphism (AFLP)for investigations below the species level. Doctoral Dissertation, Georg–August–Universität zu Göttingen.

[B20] Øvstedal DO Smith RIL (2001) Lichens of Antarctica and South Georgia, a guide to their identification and ecology. Cambridge University Press.

[B21] Poulsen RS Schmitt I Søchting U Lumbsch HT (2001) Molecular and morphological studies on the subantarctic genus *Orceolina* (Agyriaceae). Lichenologist (London, England) 33(04): 323–329. 10.1006/lich.2001.0327

[B22] Rivasseau C Farhi E Compagnon E de Gouvion Saint Cyr D van Lis R Falconet D Kuntz M Atteia A Couté A (2016) *Coccomyxaactinabiotis* sp. nov. (Trebouxiophyceae, Chlorophyta), a new green microalga living in the spent fuel cooling pool of a nuclear reactor. Journal of Phycology 52(5): 689–703. 10.1111/jpy.12442 27470701

[B23] Schmidle W (1901) Ueber drei Algengenera. Berichte der Deutschen Botanischen Gesellschaft 19: 10–24.

[B24] Smith CW Aptroot A Coppins BJ Fletcher A Gilbert OL James PW Wolseley PA (2009) The Lichens of Great Britain and Ireland. British Lichen Society, London.

[B25] Tremouillaux-Guiller J Rohr T Rohr R Huss VAR (2002) Discovery of an endophytic alga in *Ginkgobiloba*. American Journal of Botany 89: 727–733. 10.3732/ajb.89.5.727 21665672

[B26] Tschermak-Woess E (1988) The algal partner. In: Galum M (Ed.) CRC Handbook of Lichenology (Vol. 1). CRC Press, Florida.

[B27] Wirth V Hauck M Schultz M (2013) Die Flechten Deutschlands. 2 Volumes. Eugen Ulmer, Stuttgart.

[B28] Wirtz N Lumbsch HT Green TGA Turk R Pintado A Sancho L Schroeter B (2003) Lichen fungi have low cyanobiont selectivity in maritime Antarctica. The New Phytologist 160(1): 177–183. 10.1046/j.1469-8137.2003.00859.x 33873530

[B29] White TJ Bruns T Lee S Taylor J (1990) Amplification and direct sequencing of fungal ribosomal RNA genes for phylogenetics. In: Innis MA Gelfand DH Sninsky JJ White TJ (Eds) PCR Protocols.A Guide to Methods and Applications, Academic Press, San Diego, 315–322. 10.1016/B978-0-12-372180-8.50042-1

[B30] Yang QH Zhang BZ Li M Meng S (2013) Analysis of weather and sea ice at the Antarctic Great Wall Station in 2012. Chinese Journal of Polar Research 25(3): 268–277. 10.3724/SP.J.1084.2013.00268

[B31] Zoller S Lutzoni F (2003) Slow algae, fast fungi: Exceptionally high nucleotide substitution rate differences between lichenized fungi *Omphalina* and their symbiotic green algae*Coccomyxa*. Molecular Phylogenetics and Evolution 29(3): 629–640. 10.1016/S1055-7903(03)00215-X 14615198

[B32] Zuykov M Belzile C Lemaire N Gosselin M Dufresne F Pelletier E (2014) First record of the green microalgae *Coccomyxa* sp. in blue mussel *Mytilusedulis* (L.) from the Lower St. Lawrence Estuary (Québec, Canada). Journal of Invertebrate Pathology 120: 23–32. 10.1016/j.jip.2014.05.001 24837974

