# Supplementary material for: Coccomyxagreatwallensis sp. nov. (Trebouxiophyceae, Chlorophyta), a lichen epiphytic alga from Fildes Peninsula, Antarctica
Source: PhytoKeys. 2018 Nov 2;(110):39–50. doi: 10.3897/phytokeys.110.26961 (PMC6236199; doi:10.3897/phytokeys.110.26961)
Supplement: Supplementary material 3 — Table S3. Comparison of the closely related species [file phytokeys-110-039-s003.docx]

Supplementary Table S3 Comparison of the closely related species

| species | Cell size (µm) | ecology | geography | Pairwise distance (S.E.)  ITS rDNA | Pairwise distance (S.E.)  SSU rDNA |
| --- | --- | --- | --- | --- | --- |
| *Coccomyxs antarctica* | (4-7)*(8-12) | Lichen epiphyte  (*Usnea aurantiacoatra*) | Antarctica | 0.062 ± 0.013 | 0.002 ± 0.001 |
| *C. arvernensis* | (3-4)*(6-8) | Photobions of bark lichens | No record in Antarctica | 0.022 ± 0.007 | 0.001 ± 0.001 |
| *C. greatwallensis* sp. *nov.* | (3-5)*(6-12) | Lichen epiphyte  (*Psoroma hypnorum*) | Antarctica | / | / |
| *C. viridis* | (1.8-3.6)*(4.7-8.4) | Photobions of bark lichens | No record in Antarctica | 0.040 ± 0.011 | 0.001 ± 0.001 |
